# Exploratory validation of relationship functioning and non-pregnant partner behavior scales in pregnant people living with HIV in Mpumalanga Province, South Africa

**DOI:** 10.1080/16549716.2023.2210882

**Published:** 2023-05-12

**Authors:** Daniel E Sack, Tshegofatso M Seabi, Michael B Frisby, Matthew A Diemer, Godfrey H Ndlovu, Ryan G Wagner, Carolyn M Audet

**Affiliations:** a Vanderbilt Institute of Global Health, Vanderbilt University Medical Center, Nashville, TN, USA; b Medical Research Council/Wits Rural Public Health and Health Transitions Research Unit (Agincourt), School of Public Health, Faculty of Health Sciences, University of the Witwatersrand, Johannesburg, South Africa; c Department of Educational Policy Studies and the Research, Measurement, and Statistics Program, College of Education and Human Development, Georgia State University, Atlanta, GA, USA; d School of Education, University of Michigan, Ann Arbor, MI, USA

**Keywords:** HIV, sexual partners, factor analysis, interpersonal relations, South Africa, pregnancy

## Abstract

Partner engagement in antenatal care can improve care for pregnant people living with HIV. However, concerns about engaging unsupportive non-pregnant partners warrant further study to avoid engaging partners who pressure their pregnant partner to refuse testing or treatment and/or perpetuate intimate partner violence. We adapted established relationship functioning and partner behaviour questionnaires among pregnant people living with HIV initiating antenatal care in rural South Africa. We identified 13 previously validated psychometric scales with 255 items that assess relationship functioning and partner behaviour, but, to our knowledge, had not been used in Southern Africa. After item translation and cognitive interviewing with 30 pregnant people, we recruited an additional 208 pregnant people living with HIV receiving antenatal care. We conducted an exploratory factor analysis with maximum-likelihood extraction and oblique promax rotation with the 58 items and 10 scales that remained after translation and cognitive interviewing. We used parallel analysis, scree plots, and the Kaiser criterion to guide factor retention and assessed internal factor consistency via Cronbach’s alpha. Of the 208 participants recruited, 197 (95%) answered each question and were included in the analysis. Exploratory factor analysis revealed 7 factors that assessed partner social support, sexual relationship power, emotional intimacy, threatened or enacted violence, sexual intimacy, violence in relationships, and partner engagement in pregnancy care via 37 items. Factor absolute Spearman correlations ranged from 0.012 to 0.518 and Cronbach’s alpha ranged from 0.84 to 0.92. This preliminary analysis will guide further scale development. Future developments will also include relevant clinical outcomes to assess the predictive validity of the resulting measures. These steps will further refine these questions into a succinct screening tool to assess relationship functioning and partner behaviour. This screening tool may eventually guide the selection of partner-based interventions during pregnancy to improve outcomes for pregnant people and their partners.

## Introduction

In sub-Saharan Africa, interventions to increase partner engagement in antenatal care lead to increased maternal HIV testing, antiretroviral therapy adherence, retention in care, hospital delivery, and decreased parent-to-child HIV transmission [1–4]. While partner engagement interventions are often successful [3–7], concerns about engaging difficult partners persist. ‘Difficult’ partners include those who pressure their partner to refuse HIV testing or treatment, stigmatise, threaten to disclose their HIV status, and/or perpetuate intimate partner violence [5,6]. Engaging unsupportive partners can increase testing and treatment refusal and treatment abandonment, despite extensive couple-based counselling [7].

Given the potential negative outcomes associated with unsupportive non-pregnant partner involvement, it is essential that researchers and practitioners can assess relationship functioning and partner behaviour – such as intimacy, relationship satisfaction, intimate partner violence, and so on – among pregnant people living with HIV to help inform whether and/or how to engage partners in antenatal care. The length of existing scales assessing these constructs, along with uncertainty regarding how well they assess partner characteristics in regions outside where they were developed, such as sub-Saharan Africa, make relationship assessment during clinical appointments challenging. This is especially relevant given the high prevalence of HIV and intimate partner violence in the region, particularly among pregnant people living with HIV [8], in the context of an increased interest in engaging couples in antenatal care [3–7].

This adaptation and elimination of items from established relationship functioning and partner behaviour questionnaires functions as the first steps to create a short relationship functioning scale that could be used to identify potentially difficult partners among pregnant people living with HIV initiating antenatal care in rural South Africa.

## Methods

### Study site and population

We recruited 238 pregnant people with HIV receiving antenatal care at Tintswalo Hospital. Tintswalo is a district hospital in Acornhoek, located in Mpumalanga province, South Africa. Acornhoek is peri-urban and comprised of roughly 34,000 individuals, most of whom are xiTsonga-speaking [9].

### Questionnaire adaptation

We identified 13 validated scales with 255 questions assessing different relationship functioning and partner behaviour components (see Tables S1–S12 and Figure S1, for detailed descriptions of each scale) [10–29]. These scales did not represent all possible scales, but rather focused on issues identified as important to women in the area, including intimate partner violence, communication, partner support, and relationship commitment. We worked with three translators to forward and back translate each question from English to xiTsonga. Questions that did not translate well (i.e. did not fit the local context), were confusing, or were indistinguishable from other questions on the same scale after translation were deleted. Additionally, two scales (one with a picture that was difficult to adjust to the local context and another with only ‘yes’ or ‘no’ answer choices similar to questions on other scales with more variable answer choices) [10,28] were deleted, resulting in 11 scales with 89 questions [11–27]. We subsequently conducted two rounds of cognitive interviews with 30 total pregnant people attending antenatal care services at Tintswalo hospital prior to conducting interviews with the remaining 58 items from 10 scales with answer choices modified so that they were all coded on the same scale and direction to simplify the analysis.

### Participant recruitment and data collection

We recruited pregnant people living with HIV attending antenatal care at Tintswalo Hospital from 1 October 2021, to 1 March 2022. We aimed to consent 200 participants to afford an exploratory factor analysis solution to converge [30,31]. Admittedly, this sample size might provide the lower bound of adequate statistical power, pending simplicity of the factor solution and strength of item loadings [32,33]. Author GHN recruited participants, consented them, and guided them through the survey after their clinical visit. All surveys were done face-to-face with an interviewer fluent in participants’ preferred language to ensure participant understanding and assist those with low literacy. GHN read the demographic (Table S19) and scale questions aloud and recorded the participant’s responses on a tablet running the REDCap© application [34].

### Analysis plan

We conducted an exploratory factor analysis with maximum-likelihood extraction [*factanal()*] and oblique promax rotation [*rotation = ‘promax’*] in *R Statistical Software* (version 4.1.0) (code and supplement at https://github.com/dannysack/rel_func_val_sa) [30,31,35]. We used a scree plot and parallel analysis, which uses Monte Carlo Simulations to compare a simulated dataset eigenvalues to the observed dataset eigenvalues to more precisely guide factor retention [31]. Items were selected for a factor if their loading was >0.40 and the question subject matter aligned with the other questions that loaded on that factor. Two Revised Conflict Tactics Scale questions (‘I went to a doctor because of a fight with my partner’ and ‘I needed to see a doctor because of a fight with my partner, but I didn’t’) generated identical responses among all included participants, so we removed the latter, because it was more complicated. We then examined participant response distributions across factors. Cronbach’s alpha was calculated for factors to estimate internal consistency. Due to logistical barriers, we were not able to collect the desired clinical outcomes to assess predictive validity (completed antenatal visits, partner-attended antenatal visits, and HIV viral load at birth).

## Results

In each cognitive interview round (5 pregnant people to ~ 30 questions per round such that 15 pregnant people were included in each round), xiTsonga-speaking interviewers assessed comprehension, identified words and/or concepts that could be localised, and detected differences in perception of certain characteristics of 89 previously validated relationship functioning and non-pregnant partner behaviour survey items. Cognitive interviewing resulted 31 items being removed and additional minor modifications to existing questions. On the Revised Conflict Tactics Scale, each item became a two-part question that first asked if someone had experienced a negative behaviour from their partner – such as being called ugly – in the last year and, if yes, how frequently it occurred (Table 2) [22].

We then recruited 208 participants for validation and adaption of the remaining 58 items from 10 scales (Table S13–S18) [11–15,18–27]. Most (197; 95%) provided complete responses to all ten tested psychometric scales and were included in the analysis (Table 1). They reported a median age of 31 years (interquartile range [IQR] 27–36 years) and a median of 12 years of education (IQR 11–12 years). Participants were most often unmarried, but living with their partner (*n* = 62, 31.5%), married, living with their partner (*n* = 59, 29.9%), or unmarried and living without their partner (*n* = 52, 26.4%). They reported a median of 45 months with their partner (3.75 years, IQR 28–69 months), a median of one previous pregnancy (IQR 0 to 2 pregnancies), and a median of 48 months living with diagnosed HIV (IQR 24–67 months). Most of the participants (*n* = 133, 67.5%) reported that their current pregnancy was unintended.

**Table 1.** Participant demographics (*n* = 197).

Age (years)	
Median [Q1, Q3]	31.2 [27.3, 36.4]
[Min, Max]	[0.0739, 47.8]
Gestational Age (weeks)	
Median [Q1, Q3]	7.00 [6.00, 8.00]
[Min, Max]	[5.00, 35.0]
Missing	157 (79.7%)
Education (years)	
Median [Q1, Q3]	12.0 [11.0, 12.0]
[Min, Max]	[0, 16.0]
Relationship Status	
Unmarried, no partner	10 (5.1%)
Unmarried, living without partner	52 (26.4%)
Unmarried, living with partner	62 (31.5%)
Married, living without partner	14 (7.1%)
Married, living with partner	59 (29.9%)
Time with Current Non-Pregnant Partner (months)	
Median [Q1, Q3]	45.0 [28.0, 69.0]
[Min, Max]	[4.00, 248]
Previous Pregnancies	
Median [Q1, Q3]	1.00 [0, 2.00]
[Min, Max]	[0, 12.0]
Pregnancy Planned	
Yes	64 (32.5%)
No	133 (67.5%)
Time Living with HIV (months)	
Median [Q1, Q3]	48.0 [24.0, 67.0]
[Min, Max]	[1.00, 180]

Parallel analysis suggested we retain 39 of 58 items across eight factors. Items loaded onto factors with other items from their original scale, save factor 1 (see Table 2). We did not consider factor 8 because, after removing a cross-loading item, it only included one item. That revealed a final set of seven unique factors explaining 47% of the cumulative variance and containing 37 items with factors comprised of four (factor 4) to nine (factor 2) items. Factor 1 contained items that assessed partner social support, factor 2 sexual relationship power, factor 3 emotional intimacy, factor 4 threatened or enacted violence, factor 5 sexual intimacy, factor 6 violence in relationships, and factor 7 contained items that assessed partner engagement in pregnancy care.

**Table 2.** Final factor structure.

	Factor Loading^a^						
Factor Number	1	2	3	4	5	6	7
Proportion Variance	0.08	0.07	0.07	0.07	0.06	0.06	0.06
Cronbach’s alpha	0.84	0.84	0.88	0.92	0.86	0.86	0.85
**Partner Social Support**							
It helps to turn to my partner in times of need	0.51						
Do you believe you could turn to your partner if needed: to talk about a personal problem^b^	0.93						
Do you believe you could turn to your partner if needed: for advice making a decision^b^	0.96						
Do you believe you could turn to your partner if needed: for help taking care of the children^b^	0.71						
Do you believe you could turn to your partner if needed: for assistance accessing health care^b^	0.84						
**Sexual Relationship Power**							
Most of the time when we’re together, we do what my partner wants to do		0.65					
My partner has more say than I do about important decisions that affect us		0.61					
My partner tells me who I can spend time with		0.62					
I would like to leave my partner, but I do not have anywhere else to go		0.56					
My partner does what he wants, even if I do not want him to		0.68					
I am more committed to our relationship than my partner is		0.65					
When my partner and I disagree, he gets his way most of the time		0.70					
My partner always wants to know where I am		0.68					
I cannot afford to leave my partner, financially		0.48					
**Emotional Intimacy**							
I mostly feel emotionally connected with my partner			0.67				
It seems that my partner mostly feels emotionally connected with me			0.78				
My partner seems available when I need him/her emotionally			1.05				
Most of the time, my partner seems aware of my emotions, whether positive or negative			0.83				
**Threatened or Enacted Violence**							
My partner threatened to hit or throw something at me^c^				0.57			
My partner used physical force that made me fear for my life (used a weapon, choked me, etc.)^c^				0.80			
I had a sprain, bruise, or small cut because of a fight with my partner^c^				1.04			
I went to a doctor because of a fight with my partner (severe)^c^				0.99			
**Sexual Intimacy**							
Most of the time, I want to have sex when my partner also wants sex					0.79		
I am open to talk about sex with my partner					0.78		
Most of the time, my partner seems to want to have sex when I also want sex					0.77		
My partner seems to care about my sexual pleasure, not just their own					0.71		
My partner seems open to talk about sex with me					0.76		
**Violence in Relationships**							
Would you be in trouble if your partner came home and you were not there?						0.68	
Have you been forced to have sex with your partner?						0.70	
Would your partner beat you if he thought you were with someone else?						0.86	
Would your partner beat you if you went somewhere without telling him?						0.91	
Does your partner ever get angry in such a way that he hits you?						0.65	
**Partner Engagement in Pregnancy Care**							
During this pregnancy, how often has your romantic partner…Encouraged you to deliver/give birth at a clinic?^d^							0.65
During this pregnancy, how often has your romantic partner…Reminded you to take your HIV medication?^d^							1.00
During this pregnancy, how often has your romantic partner…Reminded you to go for HIV care?^d^							0.96
During this pregnancy, how often has your romantic partner…Collected medication for you or the baby from the clinic/dispensary?^d^							0.58
During this pregnancy, how often has your romantic partner…If not for COVID-19, would he come with you into the consultation room during health care visits?^d^							0.44

Note: In Factor 3, 6, and question 1 in factor 1 the answers are coded as ‘Strongly Disagree’ (0), ‘Disagree’ (1), ‘Neither Agree nor Disagree’ (2), ‘Agree’ (3), and ‘Strongly Agree’ (0). The coding is flipped in Factor 2 such that ‘Strongly Agree’ is coded as 0 and ‘Strongly Disagree’ is coded as 4. Table S22 contains the xiTsonga translations for each item.

^a^Items were selected for a factor if their loading was > 0.40 and the question subject matter aligned with the other questions that loaded on that factor.

^b^The other questions in Factor 1 have answer choices coded as ‘Definitely not’ (0), ‘No’ (1), ‘Not sure’ (2), ‘Yes’ (3), and ‘Definitely yes’ (4).

^c^Questions in Factor 4 are asked in two phases. In phase 1 they are asked: ‘Has your partner done any of the following.’ The answer choices are No or Yes. If they answer No, they are automatically coded as ‘This had never happened’ (0), whereas, if they answer Yes in Phase 1, they move on the Phase 2, where they are asked: ‘Please mark how many times the following have happened to you with your current partner.’ They can then select ‘This has happened once’ (1), ‘This has happened a few times (2)’, ‘This happens monthly’ (3), or ‘This happens weekly or more frequently’ (4).

^d^Questions in Factor 7 have answer choices coded as ‘Never’ (0), ‘Rarely’ (1), ‘Occasionally’ (2), ‘Most of the time’ (3), and ‘All of the time’ (4).

**Figure 1.**
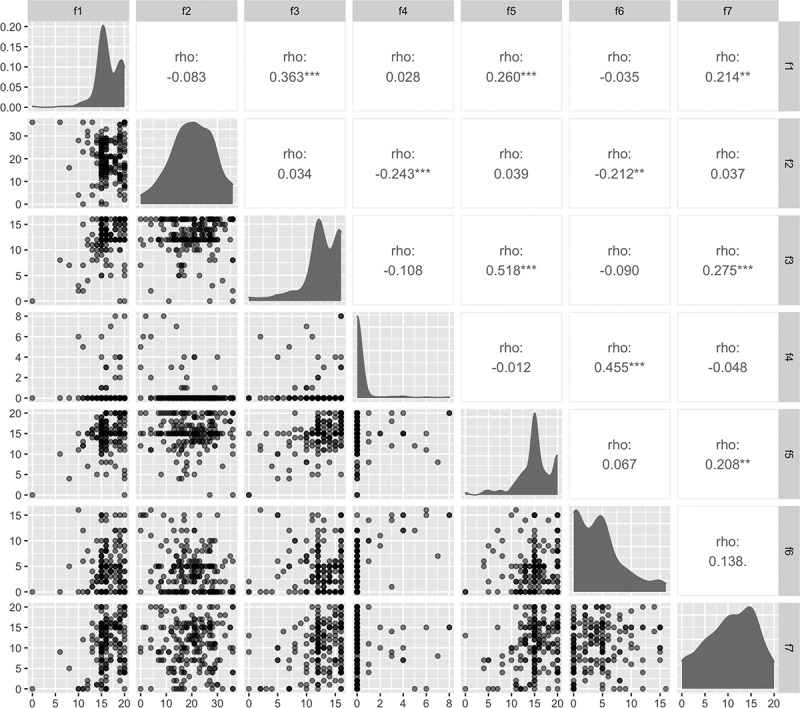
Factor distributions, relative distributions, and correlations.

Cross loadings suggested that each factor represented a unique construct (Table S20). Absolute Spearman correlations between factors ranged from 0.012 (factors 4 and 5) to 0.518 (factors 3 and 5; Figure 1) and Cronbach’s alpha ranged from 0.84 (factors 1 and 2) to 0.92 (factor 4; Table 2). Factor 4, which assessed threatened or enacted violence, generated the least variable score distribution across the interviewed participants (Figure 1).

Factor 1 (f1) assesses partner social support, f2 assesses sexual relationship power, f3 assesses emotional intimacy, f4 assesses threatened or enacted violence, f5 assesses sexual intimacy, f6 assesses violence in relationships, and f7 assesses partner engagement in pregnancy care. The lower half of plots show the distribution of scores on each factor relative to each other, with the diagonal plots showing the distribution on each factor. The upper half shows the Spearman’s rho correlation coefficient between the two factors, with * indicating a p-value < 0.05, ** indicating a p-value < 0.01, and *** indicating a p-value < 0.001.

## Discussion and conclusion

In this preliminary analysis, we were able to adapt 13 psychometric scales made up of 255 questions to 7 factors made up of 37 questions relevant to xiTsonga-speaking pregnant people living with HIV attending antenatal care appointments in rural, northeastern South Africa. This preliminary analysis will guide further scale development with a larger sample size. Specifically, confirmatory factor analysis will use new data to ‘confirm’ the 7-factor structure from EFA, and item response models can evaluate each items’ contribution to their respective scales [36]. The larger sample size can provide statistical power for estimating the large number of coefficients in these models. This additional development will add rigour to future analyses (e.g. structural equation modelling) that place these scales in relation to other variables. We will also collect relevant clinical outcomes to assess the predictive validity of the resulting measures. Such an analysis will further refine these questions into a succinct screening tool to assess relationship functioning and partner behaviour. We plan to test the value of employing this tool to guide the selection of a partner-based intervention during pregnancy to improve outcomes for pregnant people and their partners.

## Supplementary Material

Supplemental Material

## Paper context

While many studies have assessed the efficacy of partner-interventions during pregnancy for couples living with HIV, we are not aware of any that tailor interventions to each couple’s relationship characteristics. This preliminary study starts the process of developing a scale to assess relationship functioning and partner behaviour. This may eventually guide the selection of partner-based interventions during pregnancy to improve outcomes for pregnant people and their partners.
