# Supplementary material for: Exploratory validation of relationship functioning and non-pregnant partner behavior scales in pregnant people living with HIV in Mpumalanga Province, South Africa
Source: Glob Health Action. 2023 May 12;16(1):2210882. doi: 10.1080/16549716.2023.2210882 (PMC10184612; doi:10.1080/16549716.2023.2210882)
Supplement: Supplemental Material [file ZGHA_A_2210882_SM2755.docx]

**Adapted Scales Descriptions**

**Marital Conflicts Scale** (Boostanipoor and Zaker 2016):[1] Among 270 people in Tehran, Iran (155 women and 115 men), they validated eight factors with a Varimax rotation from 54 items to assess overall marital conflict. Their final scale includes subscales with the following names and Cronbach’s alpha: decreased coordination (0.81 – 5 items), decreased sexual intercourse (0.61 – 5 items), increased emotional reactions (0.70 – 8 items), increased children’s protection (0.33 – 5 items), decreased family relations with the spouse’s kindred and friends (0.89 – 6 items), increased personal relations with own family (0.86 – 6 items), separated finances (0.71 – 6 items), and decreased effective relation (0.69 – 11 items). Overall 52 of 54 items loaded. The goodness of fit and correlations between factors are shown below:


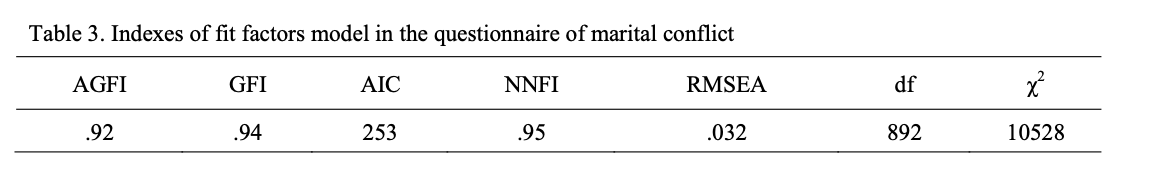


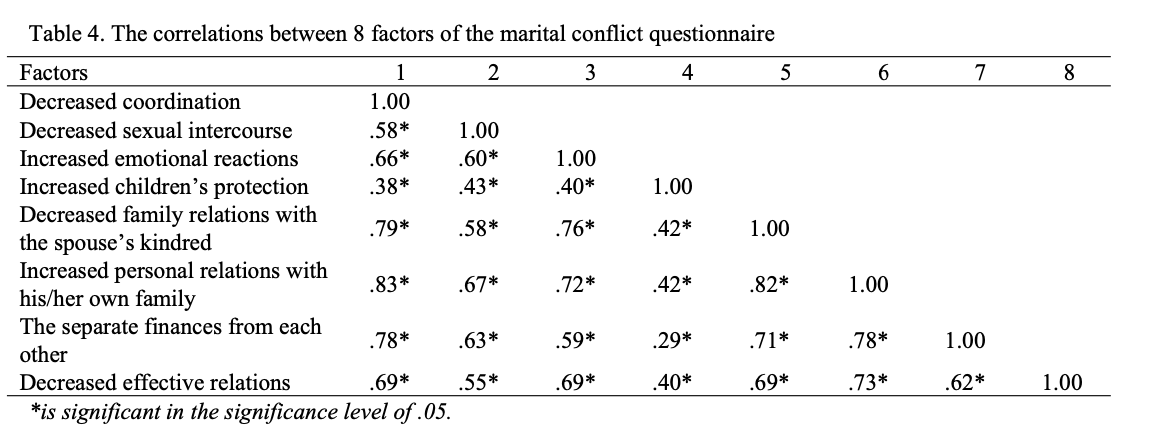


**Communication Patterns Questionnaire** (Crenshaw et al. 2017):[2] The communication patterns questionnaire assesses couples’ communication behavior, particularly related to the demand/withdrawal interaction pattern. This scale was originally developed in in the 1980s and has undergone several iterations over time. This most recent version was validated by exploratory (Promax rotation) and confirmatory factor analysis (Bayesian structural equation modelling) across four independent samples of heterosexual couples across the United States (605 total couples). The complete scale includes 35 Likert scale questions from 1 (very unlikely) to 9 (very likely), four of which assessing behavior when a problem arises, 18 that assess behavior during discussion of a problem, and 13 that assess behavior after the discussion. They found a three-factor solution the most likely, which included a constructive communication (9 items, Cronbach’s alpha range 0.720-0.863 for women and 0.666-0.885 for men), self-demand/partner-withdraw (7 items, Cronbach’s alpha range 0.770-0.813 for women and 0.617-0.805 for men), and partner-demand/self-withdraw (7 items, Cronbach’s alpha range 0.726-0.821 for women and 0.736-0.820 for men) subscales, all of which were similar for women and men. This resulted in a 23-item, 3 factor final scale.

**Multidimensional Scale of Perceived Social Support** (Zimet et al. 1988 and Eker & Arkar 1995):[3,4] The Multidimensional Scale of Perceived Social Support (MSPSS), originally developed in the United States, assesses perceived social support from family, friends, and a significant other (4 items each) with Cronbach’s alphas ranging from 0.79 to 0.98 across various samples. Each question has a Likert-type scale answer from 1 (strongly disagree) to 7 (strongly agree). Eker and Arkar re-validated the scale in Turkey, first in undergraduates and then in patients in clinics and hospitals associated with their academic center. They found Cronbach’s alphas ranging between 0.77 and 0.92 across samples and scale and associations between -0.01 and -0.41 between scales and the Beck Depression Index (-0.41 to -0.55 in patients seeking inpatient psychiatric care).

**Male Partner Involvement in Prevention of Mother-to-Child HIV Transmission** (Hampanda et al. 2020):[5] Hampanda et al. constructed a 10-item, 2-factor scale (male encouragement/reminders and active participation) to assess male involvement in the prevention of maternal-to-child transmission of HIV in pregnant and postpartum women. This scale was tested on 200 WLHIV on antiretroviral treatment at least 12 months postpartum with a current male partner in southwestern Kenya. Each item asks women to comment on an attribute of their male partner’s involvement on a 6-point Likert-like scale from never (0) to all of the time (5). After examining scree plots of principal components analysis and orthogonal rotation and assessment of item-loading, they decided on a 2-factor structure. The male encouragement/reminder factor includes 7 items (Cronbach’s alpha 0.90) and the active participation factor includes 3 items (Cronbach’s alpha 0.70) related to male behaviors. The subscale’s has a correlation coefficient of 0.56. The complete 10-item scale was associated with decreased internalized HIV stigma, increased relationship satisfaction (associated with both subscales as well), and increased HIV disclosure among couples (associated with both subscales as well). **Relationship Assessment Scale** (Hendrick 1988):[6] Hendrick developed a brief, 7-item, 1 factor scale to measure relationship satisfaction among 182 couples “in love” or in ongoing relationships in the United States. Each item is scored on a scale from 1 (low satisfaction) to 5 (high satisfaction) and items 4 and 7 are reverse scored. Principal components factor analysis resulted all 7 items loading on the same factor (Cronbach’s alpha 0.86) and the scale correlated as expected with the Love Attitudes, Sexual Attitudes, and Dyadic Adjustment scale constructs.

**Perceived Criticism** (Hooley and Tisdale 1989 and Masland and Hooley 2015):[7,8] Perceived criticism, a measure of the perceived level of criticism in one’s most meaningful relationship, has emerged as, “a robust and transdiagnostic predictor of negative clinical outcomes and relapse” across psychiatric disorders after initially predicting depression relapse in patients in Oxford, England. The scale consists for 4 items scored from 1 (not at all) to 10 (very critical) and, although there was some difficulty validating the measure in Egypt (mostly due to participant refusal to rate their partners) or when changing the scale from 1-3 (low, moderate, high), the 10-point scale has construct validity across depression, substance abuse, anxiety disorders, and psychosis. It has not shown to be related to various demographic characteristics, including sex, education, or race or ethnicity. The key question is question 2, “How critical do you think your partner is of you?”

**Sexual Relationship Power Scale** (Pulerwitz et al. 2000):[9,10] The Sexual Relationship Power Scale (SRPS) includes a Relationship Control (15 items, Cronbach’s alpha of 0.86) and Decision-Making Dominance (8 items, Cronbach’s alpha 0.62) sub-scale, both of which were initially developed in 380 English and Spanish-speaking women at an urban community health center in the United States. Oblique factor analysis eventually revealed two factors with 23 total items. Women who reported higher relationship satisfaction reported higher SRPS scores. Items on the Relationship Control subscale are scored from 1-4 from “strongly agree” to “strongly disagree” and items on the Decision-Making Dominance subscale are scored from 1-3 from “your partner” to “you”. The subscales are rescaled to a range of 1-4 and the overall score is an average of the scores from each subscale (each subscale is weighed the same). The authors recommend that respondents with more than one third of missing answers on either subscale should be excluded, and those with fewer than one third of missing answers on either subscale should have the missing answers mean imputed from the same subscale.[9]

A review of the SRPS in HIV/AIDs research reports that a slimmed down scale with references to condoms removed (items 1, 2, 8, and 22) have similar Cronbach’s alphas to the original scale (Relationship Control: 0.85, Decision-Making Dominance: 0.57).[10] South African versions combined items from the SRPS with items from another scale and reported Cronbach’s alphas of 0.84 and 0.69 for a 12 and 13-item 1-factor version respectively.[11,12] Through various iterations and languages, on multiple continents, the full scale and the Relationship Control subscale have performed well, whereas the Decision-Making Dominance subscale has not performed as well, particularly among young women. The authors also comment that scale modifications generally led to lower internal reliability across both subscales and the full scale. The scale has also reliably predicted higher condom use in multiple populations.

**Revised Conflict Tactics Scale** (Straus et al. 1996):[13] The conflict tactics scales (CTS), based on conflict theory, were used on over 70,000 participants since 1972 on diverse populations in various countries to ass how partners engage in psychological and physical attacks on each other and why and how they deal with conflicts. The original CTS measured reasoning (3 items), verbal aggression (6 items) and violence (9 items) was revised (CTS2) to measure additional types of abuse and consequences of abuse – revised scales: negotiation (6 items, Cronbach’s alpha 0.86), psychological aggression (8 items, Cronbach’s alpha 0.79), physical assault (12 items, Cronbach’s alpha 0.86) – and added new scales on sexual coercion (7 items, Cronbach’s alpha 0.87)) and injury (6 items, Cronbach’s alpha 0.95) – 39 total items, versus 18 in the original scale. The revised scale was developed in 317 undergraduates in heterosexual relationships. Each question applies to the participant and the partner (78 total questions) and items are scored from 0 “this has never happened before” to 6 “more than 20 times in the past year”, and includes 7 “not in the past year, but it did happen before” where points are allocated for the midpoint of each answer choice (25 for answer 6) and 7 is scored as 1 or 0. The correlations between scales are show below by sex:


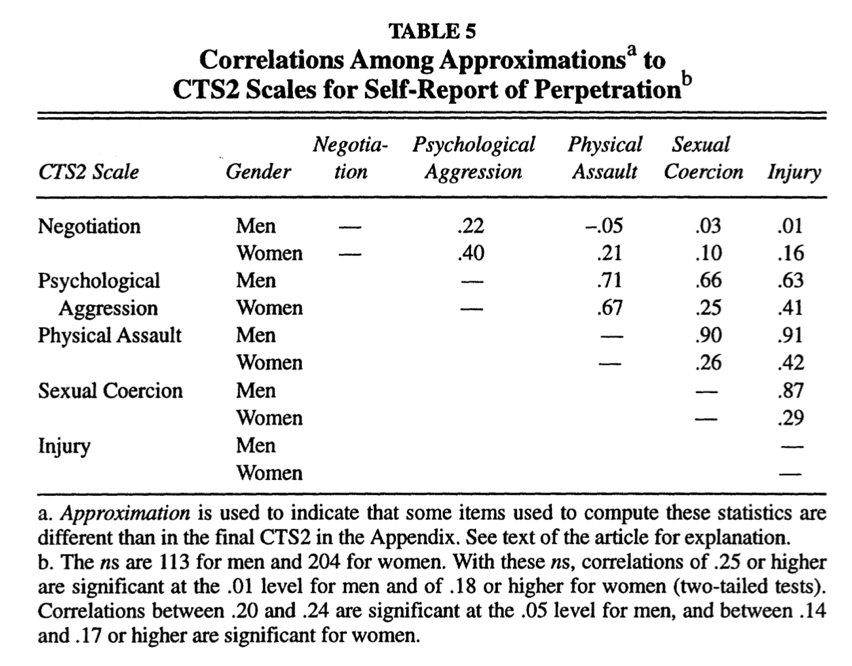


**Emotional Intimacy and Sexual Satisfaction** (Yoo et al.):[14,15] The emotional intimacy and sexual satisfaction scale was developed on 92 married couples in Ohio, in the United States. Each partner appraised their own and their partner’s emotional and sexual intimacy. There were 10 questions on Emotional Intimacy and 10 questions on Sexual Intimacy, both of which include 5 questions about the interviewee and 5 questions about the interviewee’s partner (Cronbach’s alphas from 0.61-0.87 depending on the sex of the interviewee and the scale). Responses ranged from 1 “strongly disagree” to 7 “strongly agree” on a 7-point Likert-type scale. In a validation study of 335 couples, they found that a couples’ appraisal of emotional intimacy was predictive of their sexual satisfaction through a multiple-step multiple mediator model (RMSEA = .07; NFI = .996; CFI = .997).

**Experiences in Close Relationships—Relationship Structures Questionnaire** (Fraley et al. 2011):[16] The Experiences in Close Relationships – Relationship Structures Questionnaire (ECR-RS) assesses attachment in multiple contexts (i.e. mother, father, romantic partner, best friend) and is a 9-item scale that can be applied to only one context (ECR-R, R for Revised). Items are scored on a Likert-type from 1 (strongly disagree) to 9 (strongly agree) and were validated in an online survey of 21,838 respondents in exclusive dating or martial relationships and re-validated in 388 research study participants. The 9-item scale, after varimax rotation exploratory factor analysis suggested that questions 1-6 related to avoidance (Cronbach’s alpha 0.85-0.87) and questions 7-9 relate to anxiety (Cronbach’s alpha 0.83-0.91) with a 0.38-0.44 correlation between factors within partners. Items 5 and 6 are reverse coded. Highly anxious and avoidant participants tended to be less committed per Rusbult’s Investment Model.[17]

**The Investment Model Scale – Commitment Level** (Rusbult et al. 1998):[17] The Investment Model scale rests on the Interdependence Theory, which combines the factors of level of dependence, satisfaction, quality of alternatives, and commitment level –intent to persist, long-term orientation towards, and feelings of psychological attachment in a relationship – in one scale. Through three studies, 927 introductory psychology undergraduate students in North Carolina, Rusbult et al. refined their final scale through an oblique, promax rotation factor analysis and construct validation. We use the global commitment scale, which includes 8 questions that loaded independently after refinement in the second and third iterations (Cronbach’s alpha 0.91-0.95). Items 4, 5, and 8 were negatively scored, and items are scored on a 9-point Likert-like from 0 “do not agree at all” to 8 “agree completely” (with 4 “agree somewhat”). The commitment factor was positively correlated with the Satisfaction and Investments factors and negatively correlated with the Alternatives factor. Regression analysis also revealed that the other three factors predicted the Commitment factor (R^2^ ranged from 0.69 to 0.77 in linear regression). The Commitment factor was also correlated with levels of Dyadic Adjustment and higher scores predicted individuals in relationships more likely to still be in those relationships in the next academic semester.

**Social Support and Violence in Relationship Scales** (Fifield et al. 2018):[18] Fifield et al. (2018) developed and tested two scales in a population of 443 patients with newly diagnosed with HIV in Durban, South Africa. The first scale assessed perceived social support with five questions that have answers ranging from “definitely not” (1) to “definitely yes” (5). It had a Cronbach’s alpha of 0.94 and a mean score of 17.9 (SD = 5.7) out of a total 25 points in their population. Patients with higher social support were more likely to disclose their HIV status to family and non-family members. The second scale assessed risk of violence among women in relationships (n = 236) and includes six questions with answers ranging from “strongly disagree” (1) to “strongly agree” (4), with a mean score of 12.6 (SD = 3.4) and a Cronbach’s alpha of 0.78. HIV status disclosure to a partner increased with each point increase on the violence scale.

**Inclusion of the Other in the Self (IOS)** (Aron et al. 1992 and Gächter et al. 2015):[19,20] The IOS is a single-item, pictorial measure that aims to elicit subjective closeness of relationships. It consists of seven images of two equally sized circles with linearly increasing levels of overlap that are scored from two circles that do not overlap (1) to two circles that are almost fully overlapping (7). After being validated with various other psychometric that measure relationship closeness in American undergraduates, it has also been validated among older North Americans. It is unclear how relationships conceptualized as circles translates to other populations.

**Original Survey Questions**

| **Supplemental Table 1: Marital Conflict**[1] | |
| --- | --- |
| Have any of the following issues caused problems which led to discussion about separation? | |
|  | 1. Husband's friends |
|  | 2. Wife's friends |
|  | 3. Husband's family |
|  | 4. Wife's family |
|  | 5. Husband's friends of opposite sex |
|  | 6. Wife's friends of opposite sex |
|  | 7. Husband's job |
|  | 8. Wife's job |
|  | 9. Husband's mood/temper |
|  | 10. Wife's mood/temper |
|  | 11. Husband's honesty |
|  | 12. Wife's honesty |
|  | 13. Husband's behavior at social events |
|  | 14. Wife's behavior at social events |
|  | 15. Husband's support in crisis |
|  | 16. Wife's support in crisis |
|  | 17. Religious beliefs |
|  | 18. Church attendance |
|  | 19. Charitable gifts |
|  | 20. Love and affection |
|  | 21. Sexual activities |
|  | 22. Sexual frequency |
|  | 23. Sexual fidelity |
|  | 24. Saving/investment |
|  | 25. Paying bills |
|  | 26. Spending money on clothes/hobbies |
|  | 27. Use of credit cards |
|  | 28. Long range goals and priorities |
|  | 29. Family activities |
|  | 30. Use of vacation and leisure time |
|  | 31. Resolving differences of opinion |
|  | 32. Communication |
|  | 33. Companionship |
|  | 34. Agreement upon who is "head" of household |
|  | 35. Household chores |
|  | 36. Discipline of children |
|  | 37. Intellectual discussion |
|  | 38. Gambling |
|  | 39. Alcohol/Drug use |

Could not find exact questions, these are the closest.

| **Supplemental Table 2:** **Communication Patterns Questionnaire**[2] | |
| --- | --- |
| *Constructive Communication* | |
|  | 1. When some problem in the relationship arises, both avoid discussing (-) |
|  | 2. When some problem in the relationship arises, both try to discuss |
|  | 6. During discussion of a relationship problem, both express feelings |
|  | 8. During discussion of a relationship problem, both suggest solutions and compromises |
|  | 23. After a discussion of a relationship problem, both feel understood |
|  | 24. After a discussion of a relationship problem, both withdraw (-) |
|  | 25. After a discussion of a relationship problem, both feel resolved |
|  | 26. After a discussion of a relationship problem, neither gives in (-) |
|  | 27. After a discussion of a relationship problem, both are especially nice |
| *Self-demand / Partner-withdraw* | |
|  | 3. When some problem in the relationship arises, I start discussion / P avoids |
|  | 9. During discussion of a relationship problem, I nag and demand / P withdraws |
|  | 11. During discussion of a relationship problem, I criticize / P defends |
|  | 13. During discussion of a relationship problem, I pressure to change / P resists |
|  | 17. During discussion of a relationship problem, I threaten / P gives in |
|  | 19. During discussion of a relationship problem, I call names, swear, etc. |
|  | 32. After a discussion of a relationship problem, I pressure to apologize / P resists |
| *Partner-demand / Self-withdraw* | |
|  | 4. When some problem in the relationship arises, P starts discussion / I avoid |
|  | 10. During discussion of a relationship problem, P nags and demands / I withdraw |
|  | 12. During discussion of a relationship problem, P criticizes / I defend |
|  | 14. During discussion of a relationship problem, P pressures to change / I resist |
|  | 18. During discussion of a relationship problem, P threatens / I give in |
|  | 20. During discussion of a relationship problem, P calls names, swears, etc. |
|  | 33. After a discussion of a relationship problem, P pressures to apologize / I resist |

“P” refers to partner, “(-)” refers to a negatively coded question. All questions are coded from 1-9 from “very unlikely” to “very likely”

| **Supplemental Table 3:** **Multidimensional Scale of Perceived Social Support**[3,4] | |
| --- | --- |
| *Significant Other* | |
|  | 1. There is a special person who is around when I am in need |
|  | 2. There is a special person with whom I can share my joys and sorrows |
|  | 5. I have a special person who is a real source of comfort to me |
|  | 10. There is a special person in my life who cares about my feelings |
| *Family* | |
|  | 3. My family really tries to help me |
|  | 4. I get the emotional help and support I need from my family |
|  | 8. I can talk about my problems with my family |
|  | 11. My family is willing to help me make decisions |
| *Friends* | |
|  | 6. My friends really try to help me |
|  | 7. I can count of my friends when things go wrong |
|  | 9. I have friends with whom I can share my joys and sorrows |
|  | 12. I can talk about my problems with my friends |

All questions are coded from 1-7 from “strongly disagree” to “strongly agree”

| **Supplemental Table 4:** **Male Partner Involvement in Prevention of Mother-to-Child HIV Transmission**[5] | | |
| --- | --- | --- |
| In the past year, how often has your romantic partner… | | |
|  | *Encouragement/reminders* | |
|  | | Encouraged you to deliver/give birth at a clinic? |
|  | | Reminded you to take your HIV medication? |
|  | | Reminded you to go for HIV or PMTCT care? |
|  | | Reminded you to give HIV prophylaxis medication to the baby? |
|  | | Encouraged you to feed the baby in a certain way? |
|  | | Encouraged you to take the baby for HIV testing? |
|  | | Gave you transport money to go to the clinic or dispensary? |
|  | *Active Participation* | |
|  | | Come with you to health care visits (ANC, PMTCT, or postnatal/well-child care)? |
|  | | Helped give HIV prophylaxis medication to the baby |
|  | | Collected medication for you or the baby from the clinic/dispensary? |

Response choices: 5 = all of the time, 4 = most of the time, 3 = more often than not, 2 = occasionally, 1 = rarely, 0 = never.

| **Supplemental Table 5:** **Relationship Assessment Scale**[6] | |
| --- | --- |
|  | How well does your partner meet your needs? |
|  | In general, how satisfied are you with your relationship? |
|  | How good is your relationship compared to most? |
|  | How often do you wish you hadn’t gotten in this relationship? (-) |
|  | To what extent has your relationship met your original expectations? |
|  | How much do you love your partner? |
|  | How many problems are there in your relationship? (-) |

“(-)” refers to a negatively coded question. All questions are coded from 1-5 from “low satisfaction” to “high satisfaction”

| **Supplemental Table 6:** **Perceived Criticism Scale**[7,8] | |
| --- | --- |
|  | How critical do you think you are of your male partner? |
|  | How critical do you think your partner is of you? |
|  | When your partner criticizes you, how upset do you get? |
|  | When you criticize your partner, how upset does he/she get? |

All questions are coded from 1-10 from “not at all critical” to “very critical”/“very upset” (questions 1-2/3-4)

| **Supplemental Table 7:** **Sexual Relationship Power Scale**[9] | |
| --- | --- |
| *Relationship Control** | |
|  | 1. If I asked my partner to use a condom, he would get violent |
|  | 2. If I asked my partner to use a condom, he would get angry |
|  | 3. Most of the time, we do what my partner wants to do |
|  | 4. My partner won’t let me wear certain things |
|  | 5. When my partner and I are together, I’m pretty quiet |
|  | 6. My partner has more say than I do about important decisions that affect us |
|  | 7. My partner tells me who I can spend time with |
|  | 8. If I asked my partner to use a condom, he would think I’m having sex with other people |
|  | 9. I feel trapped or stuck in our relationship |
|  | 10. My partner does what he wants, even if I do not want him to |
|  | 11. I am more committed to our relationship than my partner is |
|  | 12. When my partner and I disagree, he gets his way most of the time |
|  | 13. My partner gets more out of our relationship than I do |
|  | 14. My partner always wants to know where I am |
|  | 15. My partner might be having sex with someone else |
| *Decision-Making Dominance*** | |
|  | 16. Who usually has more say about whose friends to go out with? |
|  | 17. Who usually has more say about whether you have sex? |
|  | 18. Who usually has more say about what you do together? |
|  | 19. Who usually has more say about how often you see one another? |
|  | 20. Who usually has more say about when you talk about serious things? |
|  | 21. In general, who do you think has more power in your relationship? |
|  | 22. Who usually has more say about whether you use condoms? |
|  | 23. Who usually has more say about what types of sexual acts you do? |

*1 = Strongly Agree, 2 = Agree, 3 = Disagree, and 4 = Strongly Disagree

**1 = Your Partner, 2 = Both of You Equally, and 3 = You

| **Supplemental Table 8:** **Revised Conflict Tactics Scale**[13] | |
| --- | --- |
| Preamble: No matter how well a couple gets along, there are times when they disagree, get annoyed with the other person, want different things from each other, or just have spats or fights because they are in a bad mood, are tired or for some other reason. Couples also have many different ways of trying to settle their differences. This is a list of things that might happen when you have differences. Please mark how many times you did each to these things in the past year, and how many times your partner did them in the past year. If you or your partner did not do one of these things in the past year, but it happened before that, mark a “7" on your answer sheet for that question. If it never happened, mark an “0" on your answer sheet. | |
| *Negotiation Scale Items (subscale)* | |
|  | 1. I showed my partner I cared even though we disagreed (emotional) |
|  | 2. My partner showed care for me even though we disagreed (emotional) |
|  | **3. I explained my side of a disagreement to my partner (cognitive)** |
|  | **4. My partner explained his or her side of a disagreement to me (cognitive)** |
|  | **13. I showed respect for my partner’s feelings about an issue (emotional)** |
|  | **14. My partner showed respect for my feelings about an issue (emotional)** |
|  | 39. I said I was sure we could work out a problem (emotional) |
|  | 40. My partner was sure we could work out a problem (emotional) |
|  | **59. I suggested a compromise to a disagreement (cognitive)** |
|  | **60. My partner suggested a compromise to a disagreement (cognitive)** |
|  | 77. I agreed to try a solution to a disagreement my partner suggested (cognitive) |
|  | 78. My partner agreed to try a solution I suggested (cognitive) |
| *Psychological Aggression Items (subscale)* | |
|  | **5. I insulted or swore at my partner (minor)** |
|  | **6. My partner insulted or swore at me (minor)** |
|  | 25. I called my partner fat or ugly (severe) |
|  | 26. My partner called me fat or ugly (severe) |
|  | **29. I destroyed something belonging to my partner (severe)** |
|  | **30. My partner destroyed something belonging to me (severe)** |
|  | 35. I shouted or yelled at my partner (minor) |
|  | 36. My partner shouted or yelled at me (minor) |
|  | 49. I stomped out of the room or house or yard during a disagreement (minor) |
|  | 50. My partner stomped out of the room or house or yard during a disagreement (minor) |
|  | 65. I accused my partner of being a lousy lover (severe) |
|  | 66. My partner accused me of being a lousy lover (severe) |
|  | 67. I did something to spite my partner (minor) |
|  | 68. My partner did something to spite me (minor) |
|  | **69. I threatened to hit or throw something at my partner (severe)** |
|  | **70. My partner threatened to hit or throw something at me (severe)** |
| *Physical Assault Items (subscale)* | |
|  | 7. I threw something at my partner that could hurt (minor) |
|  | 8. My partner threw something at me that could hurt (minor) |
|  | 9. I twisted my partner’s arm or hair (minor) |
|  | 10. My partner twisted my arm or hair (minor) |
|  | **17. I pushed or shoved my partner (minor)** |
|  | **18. My partner pushed or shoved me (minor)** |
|  | 21. I used a knife or gun on my partner (severe) |
|  | 22. My partner used a knife or gun on me (severe) |
|  | **27. I punched or hit my partner with something that could hurt (severe)** |
|  | **28. My partner punched or hit me with something that could hurt (severe)** |
|  | 33. I choked my partner (severe) |
|  | 34. My partner chocked me (severe) |
|  | 37. I slammed my partner against a wall (severe) |
|  | 38. My partner slammed me against a wall (severe) |
|  | **43. I beat up my partner (severe)** |
|  | **44. My partner beat up me (severe)** |
|  | 45. I grabbed my partner (minor) |
|  | 46. My partner grabbed me (minor) |
|  | **53. I slapped my partner (minor)** |
|  | **54. My partner slapped me (minor)** |
|  | 61. I burned or scalded my partner on purpose (severe) |
|  | 62. My partner burned or scaled me on purpose (severe) |
|  | **73. I kicked my partner (severe)** |
|  | **74. My partner kicked me (severe)** |
| *Sexual Coercion Items (subscale)* | |
|  | **15. I made my partner have sex without a condom (minor)** |
|  | **16. My partner made me have sex without a condom (minor)** |
|  | 19. I use force (like hitting, holding down, or using a weapon) to make my partner have oral or anal sex (severe) |
|  | 20. My partner uses force (like hitting, holding down, or using a weapon) to make me have oral or anal sex (severe) |
|  | **47. I use force (like hitting, holding down, or using a weapon) to make my partner have sex (severe)** |
|  | **48. My partner uses force (like hitting, holding down, or using a weapon) to make me have sex (severe)** |
|  | **51. I insisted on sex when my partner did not want to (but did not use physical force) (minor)** |
|  | **52. My partner insisted on sex when I did not want to (but did not use physical force) (minor)** |
|  | 57. I used threats to make my partner have oral or anal sex (severe) |
|  | 58. My partner used threats to make me have oral or anal sex (severe) |
|  | 63. I insisted my partner have oral or anal sex (but did not used physical force) (minor) |
|  | 64. My partner insisted I have oral or anal sex (but did not used physical force) (minor |
|  | 75. I used threats to make my partner have sex (severe) |
|  | 76. My partner used threats to make me have sex (severe) |
| *Injury Items (subscale)* | |
|  | **11. I had a sprain, bruise, or small cut because of a fight with my partner (minor)** |
|  | **12. My partner had a sprain, bruise, or small cut because of a fight with me (minor)** |
|  | 23. I passed out from being hit on the head by my partner in a fight (severe) |
|  | 24. My partner passed out from being hit on the head in a fight with me (severe) |
|  | **31. I went to a doctor because of a fight with my partner (severe)** |
|  | **32. My partner went to the doctor because of a fight with me (severe)** |
|  | **41. I needed to see a doctor because of a fight with my partner, but I didn’t (severe)** |
|  | **42. My partner needed to see a doctor because of a fight with me, but didn’t (severe)** |
|  | 55. I had a broken bone from a fight with my partner (severe) |
|  | 56. My partner had a broken bone from a fight with me (severe) |
|  | 71. I felt physical pain that still hurt the next day because of a fight with my partner (minor) |
|  | 72. My partner still felt physical pain the next day because of a fight we had (minor) |

0 = This has never happened; 1= Once in the past year; 2 = Twice in the past year; 3 = 3-5 times in the past year; 4 = 6-10 times in the past year; 5 = 11-20 times in the past year; 6 = More than 20 times in the past year; 7 = Not in the past year, but it did happen before

Boldfaced questions are from the PRICS appendix (items “3/4 and 59/60”, “17/18 and 53/54”, “27/28, 43/44, and 73/74”, “29/30 and 69/70”, “31/32 and 41/42”, and “15/16 and 51/25” were combined into one question each)

| **Supplemental Table 9:** **Emotional and Sexual Intimacy Scale**[14] | | |
| --- | --- | --- |
| *Emotional Intimacy* | | |
|  | Perceptions of one’s ***own*** intimacy | |
|  |  | 1. I mostly feel emotionally connected with my partner |
|  |  | 2. I am available when my partner needs me emotionally |
|  |  | 3. I listen to and understand my partner’s emotions and feelings |
|  |  | 4. I feel comfortable with being emotionally vulnerable with my partner |
|  |  | 5. Most of the time, I am aware of my partner’s emotions, whether positive or negative |
|  | Perceptions of one’s ***partner’s*** intimacy | |
|  |  | 1. It seems that my partner mostly feels emotionally connected with me |
|  |  | 2. My partner seems available when I need him/her emotionally |
|  |  | 3. My partner seems to listen to and understand my emotions and feelings |
|  |  | 4. My partner seems comfortable with being emotionally vulnerable with me |
|  |  | 5. Most of the time, my partner seems aware of my emotions, whether positive or negative |
| *Sexual Intimacy* | | |
|  | Perceptions of one’s ***own*** intimacy | |
|  |  | 1. I am satisfied with my sex life with my partner |
|  |  | 2. Most of the time, I want to have sex when my partner also wants sex |
|  |  | 3. I care about my partner’s sexual pleasure, not just my own |
|  |  | 4. I am open to talk about sex with my partner |
|  |  | 5. I think we are a good fit as sexual partners |
|  | Perceptions of one’s ***partner’s*** intimacy | |
|  |  | 1. My partner seems satisfied with his/her sex life with me |
|  |  | 2. Most of the time, my partner seems to want to have sex when I also want sex |
|  |  | 3. My partner seems to care about my sexual pleasure, not just his/her own |
|  |  | 4. My partner seems open to talk about sex with me |
|  |  | 5. My partner seems to think that we are a good fit as sexual partners |

All questions are coded from 1-7 from “strongly disagree” to “strongly agree”

| **Supplemental Table 10:** **Experiences in Close Relationships—Revised**[16] | |
| --- | --- |
| *Anxiety* | |
|  | 1. I usually discuss my problems and concerns with this person |
|  | 2. I talk things over with this person |
|  | 3. It helps to turn to this person in times of need |
|  | 4. I find it easy to depend on this person |
|  | 5. I prefer not to show this person how I feel deep down (-) |
|  | 6. I don’t feel comfortable opening up to this person (-) |
| *Avoidance* | |
|  | 7. I’m afraid this person may abandon me |
|  | 8. I worry that this person won’t care about me as much as I care about him or her |
|  | 9. I often worry that this person doesn’t really care for me |

“(-)” refers to a negatively coded question. All questions are coded from 1-9 from “strongly disagree” to “strongly agree”

| **Supplemental Table 11:** **The Investment Model Scale**[17] | |
| --- | --- |
| *Commitment Level* | |
|  | 1. I am committed to maintaining my relationship with my partner |
|  | 2. I want our relationship to last for a very long time |
|  | 3. I feel very attached to our relationship – very strongly linked to my partner |
|  | 4. It is likely that I will date someone other than my partner within the next year (-) |
|  | 5. I would not feel very upset if our relationship were to end in the near future (-) |
|  | 6. I want our relationship to last forever |
|  | 7. I am oriented toward the long-term future of my relationship |
|  | 8. Our relationship is likely to end in the near future (-) |

“(-)” refers to a negatively coded question. All questions are coded from 0-8 from “do not agree at all” to “agree completely” (with 4 “agree somewhat”)

| **Supplemental Table 12:** **Social Support and Violence in Relationship Scales**[18] | | |
| --- | --- | --- |
| *Social Support** | | |
|  | Do you believe there are people in your life you can turn to if needed: | |
|  |  | 1. to talk about a personal problem |
|  |  | 2. to stay in bed for several weeks |
|  |  | 3. for advice making a decision |
|  |  | 4. for help taking care of the children |
|  |  | 5. for assistance accessing health care |
| *Violence in Relationship*** | | |
|  | 1. Would you be in trouble if your partner came home and you were not there? | |
|  | 2. Have you been forced to have sex with your partner? | |
|  | 3. Would your partner beat you if he thought you were with someone else? | |
|  | 4. Are you sometimes forced to do things you do not want to do? | |
|  | 5. Would your partner beat you if you went somewhere without telling him? | |
|  | 6. Does your partner ever get angry in such a way that he hits you? | |

*answers range from “definitely not” (1) to “definitely yes” (5)

**answers range from “strongly disagree” (1) to “strongly agree” (4)

**Supplementary Figure 1: The Inclusion as the Other in the Self Scale**[19]


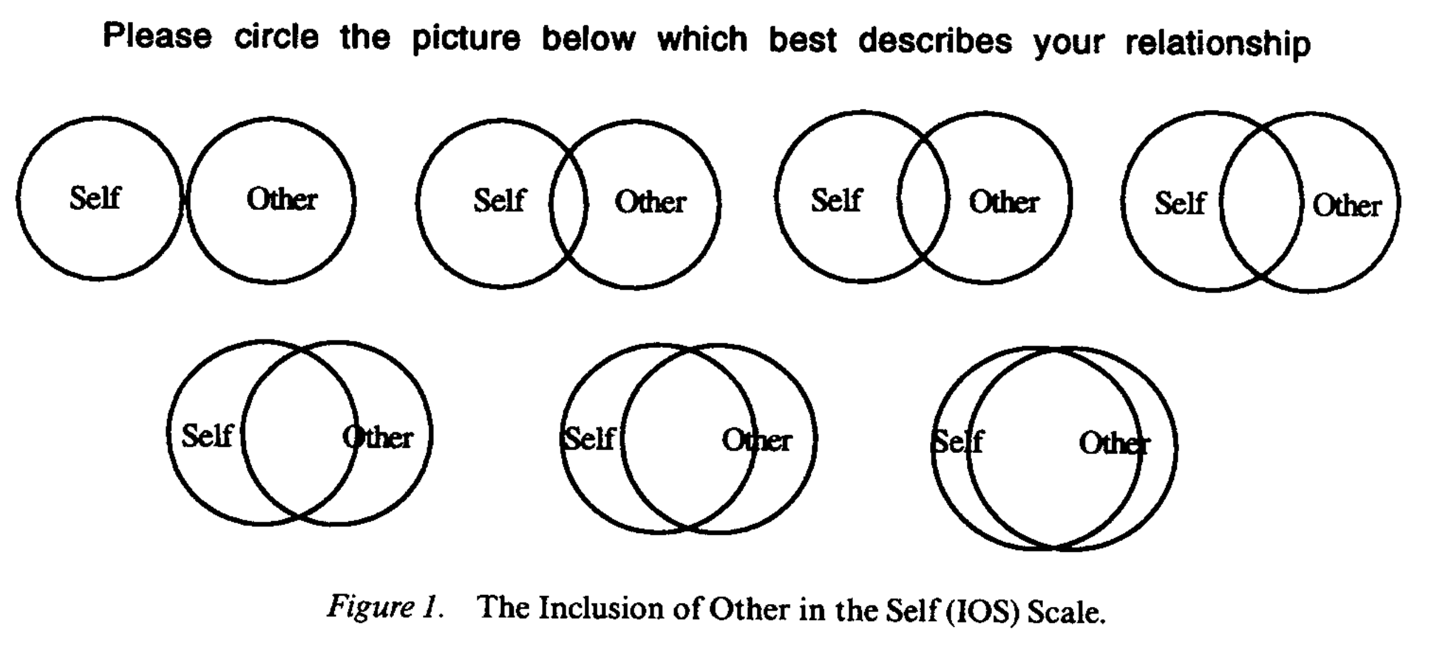


Scored from 1 (top right) to 7 (bottom left)

**Final Survey Questions**

| **Supplemental Table 13:** **Male Partner Involvement in Prevention of Mother-to-Child HIV Transmission**[5]  Please indicate the most appropriate answer. | | | | | | |
| --- | --- | --- | --- | --- | --- | --- |
| During this pregnancy, how often has your romantic partner…  Enkarhini wa kwhiri leri, ika ngaki muringani wa wena | | | | | | |
|  | *Encouragement/reminders* | | | | | |
|  | | Encouraged you to deliver/give birth at a clinic?  U hlohleteriwe ku ya kuma n’wana ekliliniki? | | | | |
|  | | Never  Aswi endleki | Rarely  Aswi talanga | Occasionally  Minkarhi yinwana | Most of the time  Minkarhi yo tala | All of the time  Minkarhi hinkwayo |
|  | | Reminded you to take your HIV medication?  U tsundzuxiwe ku teka vutshunguri bya wena bya HIV? | | | | |
|  | | Never  Aswi endleki | Rarely  Aswi talanga | Occasionally  Minkarhi yinwana | Most of the time  Minkarhi yo tala | All of the time  Minkarhi hinkwayo |
|  | | Reminded you to go for HIV care?  U tsundzuxiwe ku ya endla kambelo HIV kumbe xikalu xa vuyimani | | | | |
|  | | Never  Aswi endleki | Rarely  Aswi talanga | Occasionally  Minkarhi yinwana | Most of the time  Minkarhi yo tala | All of the time  Minkarhi hinkwayo |
|  | *Active Participation* | | | | | |
|  | | If not for COVID-19, would he come with you into the consultation room during health care visits  Loko a pfumeleriwa, kungari hi mhaka ya covid-19, ataswi kota ku ngena na wena hala mi kamberiwaka kona | | | | |
|  | | Never  Aswi endleki | Rarely  Aswi talanga | Occasionally  Minkarhi yinwana | Most of the time  Minkarhi yo tala | All of the time  Minkarhi hinkwayo |
|  | | Collected medication for you or the baby from the clinic/dispensary?  U tekile vutshunguri bya wena ku suka ekliliniki/endhawini yo hlayisela mirhi? | | | | |
|  | | Never  Aswi endleki | Rarely  Aswi talanga | Occasionally  Minkarhi yinwana | Most of the time  Minkarhi yo tala | All of the time  Minkarhi hinkwayo |

Coded from 0-4

| **Supplemental Table 14:** **Sexual Relationship Power Scale**[9]  Please indicate the most appropriate answer. | | | | | | |
| --- | --- | --- | --- | --- | --- | --- |
| *Relationship Control** | | | | | | |
|  | 3. Most of the time when we’re together, we do what my partner wants to do  Minkarhi yo tala, hi endla leswi muringani wa mina a lavaka hi endla swona | | | | | |
|  | Strongly Agree  Na pfumela swinene | | Agree  Napfumela | Neither Agree nor Disagree  Minkarhi yinwana na pfumela, minkarhi yinwana anipfumeli | Disagree  Anipfumeli | Strongly Disagree  Na ala swinene |
|  | 6. My partner has more say than I do about important decisions that affect us  Muringani wa mina u vula swo tala ku tlula mina hi swiboho swa nkoka leswi hi khumbaka ha vumbirhi | | | | | |
|  | Strongly Agree  Na pfumela swinene | | Agree  Napfumela | Neither Agree nor Disagree  Minkarhi yinwana na pfumela, minkarhi yinwana anipfumeli | Disagree  Anipfumeli | Strongly Disagree  Na ala swinene |
|  | 7. My partner tells me who I can spend time with  Muringani wa mina wa ndzi byela ku I mani loyi ndzi faneleke ndzi hungasa na yena | | | | | |
|  | Strongly Agree  Na pfumela swinene | | Agree  Napfumela | Neither Agree nor Disagree  Minkarhi yinwana na pfumela, minkarhi yinwana anipfumeli | Disagree  Anipfumeli | Strongly Disagree  Na ala swinene |
|  | NEW1. I cannot afford to leave my partner, financially  Loko swi fika eka ti mali, a ndzi nga swikoti ku sukela muringani wa mina | | | | | |
|  | Strongly Agree  Na pfumela swinene | Agree  Napfumela | | Neither Agree nor Disagree  Minkarhi yinwana na pfumela, minkarhi yinwana anipfumeli | Disagree  Anipfumeli | Strongly Disagree  Na ala swinene |
|  | NEW2. I would like to leave my partner, but I do not have anywhere else to go  Ndza swilava ku sukela muringa wa mina, mara akuna lani ndzinga yaka kona. | | | | | |
|  | Strongly Agree  Na pfumela swinene | Agree  Napfumela | | Neither Agree nor Disagree  Minkarhi yinwana na pfumela, minkarhi yinwana anipfumeli | Disagree  Anipfumeli | Strongly Disagree  Na ala swinene |
|  | 10. My partner does what he wants, even if I do not want him to  Muringani wa mina u endla leswi a lavaka swona, hambi loko ndzi nga swi lavi | | | | | |
|  | Strongly Agree  Na pfumela swinene | | Agree  Napfumela | Neither Agree nor Disagree  Minkarhi yinwana na pfumela, minkarhi yinwana anipfumeli | Disagree  Anipfumeli | Strongly Disagree  Na ala swinene |
|  | 11. I am more committed to our relationship than my partner is  Ndzi tiyimisele ngopfu eka vuxaka lebyi ku tlula leswi muringani wa mina anga tiyimiselisa xiswona | | | | | |
|  | Strongly Agree  Na pfumela swinene | | Agree  Napfumela | Neither Agree nor Disagree  Minkarhi yinwana na pfumela, minkarhi yinwana anipfumeli | Disagree  Anipfumeli | Strongly Disagree  Na ala swinene |
|  | 12. When my partner and I disagree, he gets his way most of the time  Loko mina na muringani wa mina hi ri na ku hambana, I kuma tindlela ta yena minkarhi hinkwayo | | | | | |
|  | Strongly Agree  Na pfumela swinene | | Agree  Napfumela | Neither Agree nor Disagree  Minkarhi yinwana na pfumela, minkarhi yinwana anipfumeli | Disagree  Anipfumeli | Strongly Disagree  Na ala swinene |
|  | 14. My partner always wants to know where I am  Muringani wa mina u tshamela ku lava ku tiva ku ndzi kwini | | | | | |
|  | Strongly Agree  Na pfumela swinene | | Agree  Napfumela | Neither Agree nor Disagree  Minkarhi yinwana na pfumela, minkarhi yinwana anipfumeli | Disagree  Anipfumeli | Strongly Disagree  Na ala swinene |

*Coded from 0-4

| **Supplemental Table 15:** **Revised Conflict Tactics Scale**[13]  Please indicate the most appropriate answer. | | | | | |
| --- | --- | --- | --- | --- | --- |
| **Phase 1**  Has your partner done any of the following:  Xana muringani wa wena u tshame a endla swin'wani ka leswi landzelaka:  **Phase 2**  Please mark how many times the following have happened to you with your current partner:  Kombisa leswaku leswi landzaleka swiku humelele ka ngani na muringani wa wena wa sweswi | | | | | |
| *Psychological Aggression Items (subscale)* | | | | | |
|  | 70. My partner threatened to hit or throw something at me  Muringani wa mina u ndzi chaviseterile hi ku ba kumbe a jikijela xin’wana eka mina | | | | |
|  | This has never happened  Aswise tshama swi humelela | This has happened once  Swi humelele kan'we | This has happened a few times  Swi humelerile ka ntsongo | This happens monthly  Swi humelela kanwe hi n’whetini | This happens weekly or more frequently  Swi humelele kan'we hi vhiki kumbe kakutala |
| *Physical Assault Items (subscale)* | | | | | |
|  | NEW1. My partner used physical force that could have hurt me (threw something, hit me, etc.)  Muringani wa mina u ndzi ntirhisile matimba lawa a mafenele mandzi vavisile | | | | |
|  | This has never happened  Aswise tshama swi humelela | This has happened once  Swi humelele kan'we | This has happened a few times  Swi humelerile ka ntsongo | This happens monthly  Swi humelela kanwe hi n’whetini | This happens weekly or more frequently  Swi humelele kan'we hi vhiki kumbe kakutala |
|  | NEW2. My partner used physical force that made me fear for my life (used a weapon, choked me, etc.)  Muringani wa mina u ndzi ntirhisile matimba lawa ma nga endla ndzi chavela vutomi bya mina | | | | |
|  | This has never happened  Aswise tshama swi humelela | This has happened once  Swi humelele kan'we | This has happened a few times  Swi humelerile ka ntsongo | This happens monthly  Swi humelela kanwe hi n’whetini | This happens weekly or more frequently  Swi humelele kan'we hi vhiki kumbe kakutala |
| *Injury Items (subscale)* | | | | | |
|  | 11. I had a sprain, bruise, or small cut because of a fight with my partner (minor)  Ndzi ve na ku tshinyeka, mafela ngati kumbe ku tsemiwa ku tsongo hikwalaho ko lwa na muringani wa mina | | | | |
|  | This has never happened  Aswise tshama swi humelela | This has happened once  Swi humelele kan'we | This has happened a few times  Swi humelerile ka ntsongo | This happens monthly  Swi humelela kanwe hi n’whetini | This happens weekly or more frequently  Swi humelele kan'we hi vhiki kumbe kakutala |
|  | 31. I went to a doctor because of a fight with my partner (severe)  Ndzi yile eka dokodela hikwalaho ka ku lwa na muringani wa mina | | | | |
|  | This has never happened  Aswise tshama swi humelela | This has happened once  Swi humelele kan'we | This has happened a few times  Swi humelerile ka ntsongo | This happens monthly  Swi humelela kanwe hi n’whetini | This happens weekly or more frequently  Swi humelele kan'we hi vhiki kumbe kakutala |

The above questions are asked in two phases. In phase 1, the answer choices are No (0) or Yes (1). If they answer No, they are automatically coded as “This had never happened” (0), whereas, if they answer Yes (1) in Phase 1, they move on the Phase 2, where they can pick between “This has happened once” (1) and “This happens weekly or more frequently” (4)

| **Supplemental Table 16:** **Emotional and Sexual Intimacy Scale**[14]  Please indicate the most appropriate answer. | | | | | | |
| --- | --- | --- | --- | --- | --- | --- |
| *Emotional Intimacy* | | | | | | |
|  | Perceptions of one’s ***own*** intimacy | | | | | |
|  |  | 1. I mostly feel emotionally connected with my partner  Ndzi titwa ndzi khumbeka e moyeni hi muringani wa mina | | | | |
|  |  | Strongly Disagree  Nakaneta swinene | Disagree  Aniswi pfumeli | Neither Agree nor Disagree  Minkarhi yinwana na pfumela, minkarhi yinwana anipfumeli | Agree  Napfumela | Strongly Agree  Na pfumela swinene |
|  | Perceptions of one’s ***partner’s*** intimacy | | | | | |
|  |  | 1. It seems that my partner mostly feels emotionally connected with me  Muringani wa mina utitwa a khumbeke ngopfu e moyen hima titwele ya mina | | | | |
|  |  | Strongly Disagree  Nakaneta swinene | Disagree  Aniswi pfumeli | Neither Agree nor Disagree  Minkarhi yinwana na pfumela, minkarhi yinwana anipfumeli | Agree  Napfumela | Strongly Agree  Na pfumela swinene |
|  |  | 2. My partner seems available when I need him/her emotionally  Muringani wa mina uva kona loko ndzi nwi lava e moyeni | | | | |
|  |  | Strongly Disagree  Nakaneta swinene | Disagree  Aniswi pfumeli | Neither Agree nor Disagree  Minkarhi yinwana na pfumela, minkarhi yinwana anipfumeli | Agree  Napfumela | Strongly Agree  Na pfumela swinene |
|  |  | 5. Most of the time, my partner seems aware of my emotions, whether positive or negative  Minkarhi yo tala muringani wa mina u tiva ku vaviseka ka mina loko ndzi tsakile na loko ndzinga tsakanga | | | | |
|  |  | Strongly Disagree  Nakaneta swinene | Disagree  Aniswi pfumeli | Neither Agree nor Disagree  Minkarhi yinwana na pfumela, minkarhi yinwana anipfumeli | Agree  Napfumela | Strongly Agree  Na pfumela swinene |
| *Sexual Intimacy* | | | | | | |
|  | Perceptions of one’s ***own*** intimacy | | | | | |
|  |  | 2. Most of the time, I want to have sex when my partner also wants sex  Minkarhi yo tala, ndzi lava ku endla swa masangu loko muringani wa mina na yena a lava ku endla swa masangu | | | | |
|  |  | Strongly Disagree  Nakaneta swinene | Disagree  Aniswi pfumeli | Neither Agree nor Disagree  Minkarhi yinwana na pfumela, minkarhi yinwana anipfumeli | Agree  Napfumela | Strongly Agree  Na pfumela swinene |
|  |  | 4. I am open to talk about sex with my partner  Ndzi ntshunxekile ku vulavula na muringani wa mina hi swa masangu | | | | |
|  |  | Strongly Disagree  Nakaneta swinene | Disagree  Aniswi pfumeli | Neither Agree nor Disagree  Minkarhi yinwana na pfumela, minkarhi yinwana anipfumeli | Agree  Napfumela | Strongly Agree  Na pfumela swinene |
|  | Perceptions of one’s ***partner’s*** intimacy | | | | | |
|  |  | 2. Most of the time, my partner seems to want to have sex when I also want sex  Minkarhi yo tala, muringani wa mina u lava ku endla swa masangu loko na mina ndzi lava swa masangu | | | | |
|  |  | Strongly Disagree  Nakaneta swinene | Disagree  Aniswi pfumeli | Neither Agree nor Disagree  Minkarhi yinwana na pfumela, minkarhi yinwana anipfumeli | Agree  Napfumela | Strongly Agree  Na pfumela swinene |
|  |  | 3. My partner seems to care about my sexual pleasure, not just their own  Muringani wa mina u vonakala ari na mhaka hi ku enereseka ka mina hi swa masangu, ku nga ri swa yena ntsena | | | | |
|  |  | Strongly Disagree  Nakaneta swinene | Disagree  Aniswi pfumeli | Neither Agree nor Disagree  Minkarhi yinwana na pfumela, minkarhi yinwana anipfumeli | Agree  Napfumela | Strongly Agree  Na pfumela swinene |
|  |  | 4. My partner seems open to talk about sex with me  Muringani wa mina u tikomba a ntshunxekile ku vulavula hi swa masangu na mina. | | | | |
|  |  | Strongly Disagree  Nakaneta swinene | Disagree  Aniswi pfumeli | Neither Agree nor Disagree  Minkarhi yinwana na pfumela, minkarhi yinwana anipfumeli | Agree  Napfumela | Strongly Agree  Na pfumela swinene |

Coded from 0-4

| **Supplemental Table 17:** **Experiences in Close Relationships—Revised**[16]  Please indicate the most appropriate answer. | | | | | |
| --- | --- | --- | --- | --- | --- |
| *Anxiety* | | | | | |
|  | 3. It helps to turn to my partner in times of need  Swa pfuna ku ta eka muringani wa mina hi minkarhi leyi u n’wi lavaka | | | | |
|  | Strongly Disagree  Nakaneta swinene | Disagree  Aniswi pfumeli | Neither Agree nor Disagree  Minkarhi yinwana na pfumela, minkarhi yinwana anipfumeli | Agree  Napfumela | Strongly Agree  Na pfumela swinene |

| **Supplemental Table 18:** **Social Support and Violence in Relationship Scales**[18]  Please indicate the most appropriate answer. | | | | | | | | | | |
| --- | --- | --- | --- | --- | --- | --- | --- | --- | --- | --- |
| *Social Support* | | | | | | | | | | |
|  | Do you believe you could turn to your partner if needed:  Xana wa tshemba leswaku ku na muringani wa wena loyi unga yaka eka yena loko uri na xilaveko: | | | | | | | | | |
|  |  | 1. to talk about a personal problem  Ku vulavula hi xiphiqo xa wena n’wini | | | | | | | | |
|  |  | Definitely not  Ani pfumeli naka tsongo | | No  Anipfumeli | | Not sure  Nakanakana | | Yes  Napfumela | | Definitely yes  Napfumela swinene |
|  |  | 3. for advice making a decision  Ku kuma xitsundzuxo xaku teka xiboho | | | | | | | | |
|  |  | Definitely not  Ani pfumeli naka tsongo | | No  Anipfumeli | | Not sure  Nakanakana | | Yes  Napfumela | | Definitely yes  Napfumela swinene |
|  |  | 4. for help taking care of the children  Mpfuneto wa ku hlayisa vana | | | | | | | | |
|  |  | Definitely not  Ani pfumeli naka tsongo | | No  Anipfumeli | | Not sure  Nakanakana | | Yes  Napfumela | | Definitely yes  Napfumela swinene |
|  |  | 5. for assistance accessing health care  Ku pfuniwa ku ya fikelela ndhawu ya vutshunguri | | | | | | | | |
|  |  | Definitely not  Ani pfumeli naka tsongo | | No  Anipfumeli | | Not sure  Nakanakana | | Yes  Napfumela | | Definitely yes  Napfumela swinene |
| *Violence in Relationship* | | | | | | | | | | |
|  | 1. Would you be in trouble if your partner came home and you were not there?  U nga va u ri khombyeni loko muringani wa wena a nga vuya kaya a kuma u nga ri kona? | | | | | | | | | |
|  | Strongly Disagree  Nakaneta swinene | | Disagree  Aniswi pfumeli | | Neither Agree nor Disagree  Minkarhi yinwana na pfumela, minkarhi yinwana anipfumeli | | Agree  Napfumela | | Strongly Agree  Na pfumela swinene | |
|  | 2. Have you been forced to have sex with your partner?  U tshame u nga sindzisiwa ku endla swa masangu na muringani wa wena? | | | | | | | | | |
|  | Strongly Disagree  Nakaneta swinene | | Disagree  Aniswi pfumeli | | Neither Agree nor Disagree  Minkarhi yinwana na pfumela, minkarhi yinwana anipfumeli | | Agree  Napfumela | | Strongly Agree  Na pfumela swinene | |
|  | 3. Would your partner beat you if he thought you were with someone else?  Muringani wa wena anga ku ba loko a nga ehleketela leswaku a wu ri na munhu un’wana? | | | | | | | | | |
|  | Strongly Disagree  Nakaneta swinene | | Disagree  Aniswi pfumeli | | Neither Agree nor Disagree  Minkarhi yinwana na pfumela, minkarhi yinwana anipfumeli | | Agree  Napfumela | | Strongly Agree  Na pfumela swinene | |
|  | 5. Would your partner beat you if you went somewhere without telling him?  A nga ku ba muringani wa wena loko u nga ya kun’wana u nga n’wi byelanga? | | | | | | | | | |
|  | Strongly Disagree  Nakaneta swinene | | Disagree  Aniswi pfumeli | | Neither Agree nor Disagree  Minkarhi yinwana na pfumela, minkarhi yinwana anipfumeli | | Agree  Napfumela | | Strongly Agree  Na pfumela swinene | |
|  | 6. Does your partner ever get angry in such a way that he hits you?  Xana muringani wa wena wa hlundzuka ku fika laha a ku baka? | | | | | | | | | |
|  | Strongly Disagree  Nakaneta swinene | | Disagree  Aniswi pfumeli | | Neither Agree nor Disagree  Minkarhi yinwana na pfumela, minkarhi yinwana anipfumeli | | Agree  Napfumela | | Strongly Agree  Na pfumela swinene | |

Coded from 0-4

| **Supplemental Table 19: Enrollment Interview Questions** | |  |
| --- | --- | --- |
| Variable | Question | |
| Name | What is your full name? | |
| Date of Birth | What is your date of birth? | |
| Gestational Age | How far along are you into your pregnancy? | |
| Years of Education | What is the highest education that you achieved? | |
| Relationship Status | What is your current relationship status? | |
| Months with Partner | How long have you been with your current male partner? | |
| Previous Pregnancies | How many times have you been pregnant before this pregnancy? | |
| Pregnancy Intention | Was this pregnancy planned? | |
| HIV Duration | How long have you known you have HIV? | |

| **Supplemental Table 20: Factor Analysis Cross Loadings** | | | | | | | | |
| --- | --- | --- | --- | --- | --- | --- | --- | --- |
|  | Factor | | | | | | | |
|  | 1 | 2 | 3 | 4 | 5 | 6 | 7 | 8 |
| st10_3: It helps to turn to my partner in times of need | **0.51** | 0.12 | 0.21 | -0.04 | 0.08 | -0.04 | -0.08 | 0.07 |
| st12_ss1: Do you believe you could turn to your partner if needed to talk about a personal problem | **0.93** | 0.04 | -0.03 | 0.00 | -0.10 | -0.07 | -0.06 | -0.04 |
| st12_ss3: Do you believe you could turn to your partner if needed for advice making a decision | **0.96** | -0.03 | -0.14 | -0.03 | -0.09 | -0.04 | -0.01 | -0.03 |
| st12_ss4: Do you believe you could turn to your partner if needed for help taking care of the children | **0.71** | -0.06 | -0.13 | 0.05 | 0.05 | -0.06 | -0.01 | -0.03 |
| st12_ss5: Do you believe you could turn to your partner if needed for assistance accessing health care | **0.84** | -0.03 | -0.23 | 0.05 | 0.04 | -0.07 | -0.03 | 0.00 |
| st7_3: Most of the time when we're together, we do what my partner wants to do | -0.14 | **0.65** | 0.03 | -0.08 | 0.00 | 0.16 | 0.12 | -0.01 |
| st7_6: My partner has more say than I do about important decisions that affect us | -0.20 | **0.61** | 0.13 | 0.02 | 0.03 | -0.02 | 0.08 | 0.02 |
| st7_7: My partner tells me who I can spend time with | -0.05 | **0.62** | -0.06 | 0.04 | 0.05 | -0.17 | 0.15 | 0.09 |
| st7_n2: I would like to leave my partner, but I do not have anywhere else to go | 0.09 | **0.56** | 0.17 | -0.13 | -0.11 | 0.21 | -0.28 | 0.02 |
| st7_10: My partner does what he wants, even if I do not want him to | 0.12 | **0.68** | 0.06 | -0.03 | -0.09 | 0.10 | 0.08 | 0.05 |
| st7_11: I am more committed to our relationship than my partner is | 0.05 | **0.65** | -0.01 | 0.09 | -0.01 | 0.18 | -0.21 | -0.09 |
| st7_12: When my partner and I disagree, he gets his way most of the time | 0.12 | **0.70** | -0.11 | 0.04 | -0.06 | 0.02 | 0.07 | -0.04 |
| st7_14: My partner always wants to know where I am | -0.01 | **0.68** | 0.03 | 0.07 | -0.02 | -0.02 | -0.07 | 0.02 |
| st9_eo1: I mostly feel emotionally connected with my partner | 0.02 | 0.00 | **0.67** | 0.06 | 0.09 | 0.00 | -0.14 | -0.01 |
| st9_ep1: It seems that my partner mostly feels emotionally connected with me | -0.17 | 0.09 | **0.78** | 0.05 | 0.10 | -0.01 | -0.03 | 0.04 |
| st9_ep2: My partner seems available when I need him/her emotionally | -0.11 | 0.03 | **1.05** | 0.09 | 0.04 | -0.10 | -0.10 | 0.08 |
| st9_ep5: Most of the time, my partner seems aware of my emotions, whether positive or negative | -0.08 | -0.06 | **0.83** | 0.05 | 0.19 | -0.11 | -0.03 | -0.02 |
| st8_70: My partner threatened to hit or throw something at me | 0.02 | -0.03 | -0.03 | **0.57** | -0.02 | 0.10 | 0.01 | 0.29 |
| st8_n1: My partner used physical force that could have hurt me (threw something, hit me, etc.) | -0.03 | 0.07 | 0.13 | **0.65** | 0.00 | -0.01 | -0.02 | **0.60** |
| st8_n2: My partner used physical force that made me fear for my life (used a weapon, choked me, etc.) | -0.08 | 0.00 | 0.15 | **0.80** | 0.00 | -0.01 | 0.02 | 0.34 |
| st8_11: I had a sprain, bruise, or small cut because of a fight with my partner | 0.02 | 0.01 | 0.06 | **1.04** | -0.03 | 0.01 | 0.01 | -0.12 |
| st8_31: I went to a doctor because of a fight with my partner | 0.04 | -0.02 | -0.01 | **0.99** | 0.03 | 0.06 | -0.01 | -0.20 |
| st9_so2: Most of the time, I want to have sex when my partner also wants sex | -0.09 | -0.02 | 0.11 | 0.02 | **0.79** | 0.05 | -0.08 | -0.10 |
| st9_so4: I am open to talk about sex with my partner | -0.14 | -0.09 | 0.11 | -0.01 | **0.78** | -0.03 | 0.13 | 0.04 |
| st9_sp2: Most of the time, my partner seems to want to have sex when I also want sex | -0.04 | 0.03 | -0.04 | -0.02 | **0.77** | 0.09 | -0.12 | 0.03 |
| st9_sp3: My partner seems to care about my sexual pleasure, not just their own | 0.07 | -0.01 | -0.12 | -0.04 | **0.71** | -0.01 | 0.12 | -0.02 |
| st9_sp4: My partner seems open to talk about sex with me | -0.02 | -0.03 | -0.01 | 0.01 | **0.76** | -0.01 | 0.07 | 0.01 |
| st12_vr1: Would you be in trouble if your partner came home and you were not there? | 0.02 | -0.02 | -0.23 | -0.14 | 0.10 | **0.68** | 0.03 | 0.09 |
| st12_vr2: Have you been forced to have sex with your partner? | -0.12 | 0.00 | -0.01 | 0.07 | 0.06 | **0.70** | 0.04 | -0.04 |
| st12_vr3: Would your partner beat you if he thought you were with someone else? | 0.00 | 0.08 | 0.00 | -0.06 | -0.05 | **0.86** | -0.06 | -0.01 |
| st12_vr5: Would your partner beat you if you went somewhere without telling him? | -0.09 | 0.10 | -0.14 | -0.04 | 0.02 | **0.91** | -0.01 | -0.01 |
| st12_vr6: Does your partner ever get angry in such a way that he hits you? | -0.15 | 0.01 | -0.01 | 0.29 | -0.04 | **0.65** | 0.03 | 0.03 |
| st4_1: During this pregnancy, how often has your romantic partner encouraged you to deliver/give birth at a clinic | -0.07 | 0.09 | 0.03 | 0.04 | 0.06 | 0.03 | **0.65** | -0.11 |
| st4_2: During this pregnancy, how often has your romantic partner reminded you to take your HIV medication | -0.01 | 0.00 | -0.22 | 0.04 | -0.01 | -0.03 | **1.00** | -0.03 |
| st4_3: During this pregnancy, how often has your romantic partner reminded you to go for HIV care | -0.08 | 0.00 | -0.06 | -0.03 | 0.04 | -0.02 | **0.96** | 0.00 |
| st4_6: During this pregnancy, how often has your romantic partner collected medication for you or the baby from the clinic/dispensary | -0.07 | 0.02 | 0.10 | -0.05 | 0.07 | 0.10 | **0.58** | 0.01 |
| st8_26: My partner called me ugly | -0.07 | -0.05 | 0.06 | 0.04 | -0.01 | 0.08 | -0.05 | **0.55** |
| st2_8: During discussion of a relationship problem, both suggest solutions and compromises | 0.04 | 0.07 | 0.16 | -0.07 | 0.07 | 0.16 | 0.13 | -0.13 |
| st3_2: I can communicate with my partner about good and bad situations | 0.18 | 0.02 | 0.11 | -0.02 | 0.08 | 0.10 | 0.14 | -0.10 |
| st3_10: My partner cares about my feelings | 0.10 | 0.10 | 0.15 | -0.01 | 0.21 | 0.09 | 0.19 | -0.07 |
| st4_4: During this pregnancy, how often has your romantic partner gave you transport money to go to the clinic or dispensary | 0.18 | -0.12 | 0.21 | -0.08 | -0.20 | 0.09 | 0.31 | 0.12 |
| st4_5: During this pregnancy, how often has your romantic partner, if not for COVID-19, would he come with you in to the consultation room during health care visits | 0.17 | 0.08 | 0.21 | 0.06 | -0.18 | -0.02 | **0.44** | 0.10 |
| st5_1: In general, how satisfied are you with your relationship? | 0.38 | -0.01 | 0.31 | -0.15 | -0.01 | -0.03 | 0.03 | -0.03 |
| st7_n1: I cannot afford to leave my partner, financially | -0.03 | **0.48** | 0.00 | -0.08 | 0.01 | -0.02 | -0.16 | -0.03 |
| st7_15: My partner might be having sex with someone else | 0.30 | 0.31 | -0.09 | 0.17 | 0.00 | -0.05 | 0.10 | -0.12 |
| st7_16: Who usually has more say about whose friends to go out with? | -0.18 | 0.29 | -0.09 | -0.06 | 0.24 | -0.24 | 0.01 | 0.03 |
| st7_17: Who usually has more say about whether you have sex? | -0.14 | 0.09 | -0.13 | -0.03 | 0.10 | -0.09 | -0.08 | 0.05 |
| st_7_18: Who usually has more say about what you do together? | 0.04 | 0.23 | -0.21 | -0.04 | 0.01 | -0.11 | 0.07 | 0.02 |
| st7_21: In general, who do you think has more power in your relationship? | -0.06 | 0.21 | -0.14 | -0.01 | -0.05 | -0.21 | 0.11 | 0.05 |
| st9_so1: I am satisfied with my sex life with my partner | 0.20 | 0.01 | 0.33 | -0.09 | 0.39 | -0.11 | -0.11 | 0.08 |
| st10_1: I usually discuss my problems and concerns with my partner | 0.36 | 0.07 | -0.02 | 0.08 | 0.40 | -0.02 | 0.08 | 0.10 |
| st10_7: I'm afraid my partner may abandon me | 0.08 | -0.12 | 0.13 | -0.01 | 0.16 | 0.14 | -0.04 | -0.06 |
| st10_9: I often worry that my partner doesn't really care for me | -0.05 | -0.16 | 0.06 | -0.01 | 0.08 | 0.16 | -0.04 | -0.02 |
| st11_1: I am committed to maintaining my relationship with my partner | 0.15 | -0.11 | 0.17 | -0.08 | -0.09 | 0.16 | 0.12 | 0.05 |
| st11_6: I want our relationship to last forever | 0.39 | -0.03 | 0.24 | -0.01 | 0.04 | 0.00 | -0.06 | -0.07 |
| st8_30: My partner destroyed something belonging to me | 0.12 | 0.01 | -0.15 | 0.14 | 0.06 | 0.27 | -0.02 | 0.30 |
| st8_36: My partner shouted or yelled at me | 0.23 | -0.06 | -0.22 | 0.09 | 0.07 | 0.20 | -0.08 | 0.15 |

Boldfaced if loading > 0.40

| **Supplemental Table 21. Final Factor Structure and Translations** |
| --- |
| **Partner Social Support** |
| It helps to turn to my partner in times of need  *Swa pfuna ku ta eka muringani wa mina hi minkarhi leyi u n'wi lavaka* |
| Do you believe you could turn to your partner if needed: to talk about a personal problem*  *Xana wa tshemba leswaku ku na muringani wa wena loyi unga yaka eka yena loko uri na xilaveko: Ku vulavula hi xiphiqo xa wena n'wini* |
| Do you believe you could turn to your partner if needed: for advice making a decision*  *Xana wa tshemba leswaku ku na muringani wa wena loyi unga yaka eka yena loko uri na xilaveko: Ku kuma xitsundzuxo xaku teka xiboho* |
| Do you believe you could turn to your partner if needed: for help taking care of the children*  *Xana wa tshemba leswaku ku na muringani wa wena loyi unga yaka eka yena loko uri na xilaveko: Mpfuneto wa ku hlayisa vana* |
| Do you believe you could turn to your partner if needed: for assistance accessing health care*  *Xana wa tshemba leswaku ku na muringani wa wena loyi unga yaka eka yena loko uri na xilaveko: Ku pfuniwa ku ya !kelela ndhawu ya vutshunguri* |
| **Sexual Relationship Power** |
| Most of the time when we're together, we do what my partner wants to do  *Minkarhi yo tala, hi endla leswi muringani wa mina a lavaka hi endla swona* |
| My partner has more say than I do about important decisions that affect us  *Muringani wa mina u vula swo tala ku tlula mina hi swiboho swa nkoka leswi hi khumbaka ha vumbirhi* |
| My partner tells me who I can spend time with  *Muringani wa mina wa ndzi byela ku I mani loyi ndzi faneleke ndzi hungasa na yena* |
| I would like to leave my partner, but I do not have anywhere else to go  *Ndza swilava ku sukela muringa wa mina, mara akuna lani ndzinga yaka kona.* |
| My partner does what he wants, even if I do not want him to  *Muringani wa mina u endla leswi a lavaka swona, hambi loko ndzi nga swi lavi* |
| I am more committed to our relationship than my partner is  *Ndzi tiyimisele ngopfu eka vuxaka lebyi ku tlula leswi muringani wa mina anga tiyimiselisa xiswona* |
| When my partner and I disagree, he gets his way most of the time  *Loko mina na muringani wa mina hi ri na ku hambana, I kuma tindlela ta yena minkarhi hinkwayo* |
| My partner always wants to know where I am  *Muringani wa mina u tshamela ku lava ku tiva ku ndzi kwini* |
| I cannot afford to leave my partner, financially  *Loko swi fika eka ti mali, a ndzi nga swikoti ku sukela muringani wa mina* |
| **Emotional Intimacy** |
| I mostly feel emotionally connected with my partner  *Ndzi titwa ndzi khumbeka e moyeni hi muringani wa mina* |
| It seems that my partner mostly feels emotionally connected with me  *Muringani wa mina utitwa a khumbeke ngopfu e moyen hima titwele ya mina* |
| My partner seems available when I need him/her emotionally  *Muringani wa mina uva kona loko ndzi nwi lava e moyeni* |
| Most of the time, my partner seems aware of my emotions, whether positive or negative  *Minkarhi yo tala muringani wa mina u tiva ku vaviseka ka mina loko ndzi tsakile na loko ndzinga tsakanga* |
| **Threatened or Enacted Violence** |
| My partner threatened to hit or throw something at me**  *Muringani wa mina u ndzi chaviseterile hi ku ba kumbe a jikijela xin’wana eka mina* |
| My partner used physical force that made me fear for my life (used a weapon, choked me, etc.) **  *Muringani wa mina u ndzi ntirhisile matimba lawa ma nga endla ndzi chavela vutomi bya mina* |
| I had a sprain, bruise, or small cut because of a fight with my partner**  *Ndzi ve na ku tshinyeka, mafela ngati kumbe ku tsemiwa ku tsongo hikwalaho ko lwa na muringani wa mina** |
| I went to a doctor because of a fight with my partner (severe)**  *Ndzi yile eka dokodela hikwalaho ka ku lwa na muringani wa mina* |
| **Sexual Intimacy** |
| Most of the time, I want to have sex when my partner also wants sex  *Minkarhi yo tala, ndzi lava ku endla swa masangu loko muringani wa mina na yena a lava ku endla swa masangu* |
| I am open to talk about sex with my partner  *Ndzi ntshunxekile ku vulavula na muringani wa mina hi swa masangu* |
| Most of the time, my partner seems to want to have sex when I also want sex  *Minkarhi yo tala, muringani wa mina u lava ku endla swa masangu loko na mina ndzi lava swa masangu* |
| My partner seems to care about my sexual pleasure, not just their own  *Muringani wa mina u vonakala ari na mhaka hi ku enereseka ka mina hi swa masangu, ku nga ri swa yena ntsena* |
| My partner seems open to talk about sex with me  *Muringani wa mina u tikomba a ntshunxekile ku vulavula hi swa masangu na mina.* |
| **Violence in Relationships** |
| Would you be in trouble if your partner came home and you were not there?  *U nga va u ri khombyeni loko muringani wa wena a nga vuya kaya a kuma u nga ri kona?* |
| Have you been forced to have sex with your partner?  *U tshame u nga sindzisiwa ku endla swa masangu na muringani wa wena?* |
| Would your partner beat you if he thought you were with someone else?  *Muringani wa wena anga ku ba loko a nga ehleketela leswaku a wu ri na munhu un'wana?* |
| Would your partner beat you if you went somewhere without telling him?  *A nga ku ba muringani wa wena loko u nga ya kun'wana u nga n'wi byelanga?* |
| Does your partner ever get angry in such a way that he hits you?  *Xana muringani wa wena wa hlundzuka ku !ka laha a ku baka?* |
| **Partner Engagement in Pregnancy Care** |
| During this pregnancy, how often has your romantic partner...Encouraged you to deliver/give birth at a clinic?***  *Enkarhini wa kwhiri leri, ika ngaki muringani wa wena...U hlohleteriwe ku ya kuma n'wana ekliliniki?**** |
| During this pregnancy, how often has your romantic partner...Reminded you to take your HIV medication?***  *Enkarhini wa kwhiri leri, ika ngaki muringani wa wena...U tsundzuxiwe ku teka vutshunguri bya wena bya HIV?* |
| During this pregnancy, how often has your romantic partner...Reminded you to go for HIV care?***  *Enkarhini wa kwhiri leri, ika ngaki muringani wa wena...U tsundzuxiwe ku ya endla kambelo HIV kumbe xikalu xa vuyimani?* |
| During this pregnancy, how often has your romantic partner...Collected medication for you or the baby from the clinic/dispensary?***  *Enkarhini wa kwhiri leri, ika ngaki muringani wa wena...U tekile vutshunguri bya wena ku suka ekliliniki/endhawini yo hlayisela mirhi?* |
| During this pregnancy, how often has your romantic partner...If not for COVID-19, would he come with you into the consultation room during health care visits?***  *Enkarhini wa kwhiri leri, ika ngaki muringani wa wena...Loko a pfumeleriwa, kungari hi mhaka ya covid-19, ataswi kota ku ngena na wena hala mi kamberiwaka kona?* |

In Factor 3, 6, and question 1 in factor 1 the answers are coded as “Strongly Disagree” (Nakaneta swinene; 0), “Disagree” (Aniswi pfumeli; 1), “Neither Agree nor Disagree” (Minkarhi yinwana na pfumela, minkarhi yinwana anipfumeli; 2), “Agree” (Napfumela; 3), and “Strongly Agree” (Na pfumela swinene; 0). The coding is flipped in Factor 2 such that “Strongly Agree” is coded as 0 and “Strongly Disagree” is coded as 4.

*The other questions in Factor 1 have answer choices coded as “Definitely not” (Ani pfumeli naka tsongo; 0), “No” (Anipfumeli; 1), “Not sure” (Nakanakana; 2), “Yes” (Napfumela; 3), and “Definitely yes” (Napfumela swinene; 4).

**Questions in Factor 4 are asked in two phases. In phase 1 they are asked: “Has your partner done any of the following” (Xana muringani wa wena u tshame a endla swin'wani ka leswi landzelaka). The answer choices are No (Anipfumeli) or Yes (Napfumela). If they answer No, they are automatically coded as “This had never happened” (Swi humelele kan'we; 0), whereas, if they answer Yes in Phase 1, they move on the Phase 2, where they are asked: “Please mark how many times the following have happened to you with your current partner” (Kombisa leswaku leswi landzaleka swiku humelele ka ngani na muringani wa wena wa sweswi). They can then select “This has happened once” (Swi humelele kan'we; 1), “This has happened a few times (Swi humelerile ka ntsongo; 2), “This happens monthly (Swi humelela kanwe hi n'whetini; 3), or “This happens weekly or more frequently” (Swi humelele kan'we hi vhiki kumbe kakutala; 4)

***Questions in Factor 7 have answer choices coded as “Never” (Aswi endleki; 0), “Rarely” (Aswi talanga; 1), “Occasionally” (Minkarhi yinwana; 2), “Most of the time” (Minkarhi yo tala; 3), and “All of the time” (Minkarhi hinkwayo; 4).

**References**

1. Boostanipoor A, Zaker BS. The Questionnaire of Marital Conflicts: A Confirmatory Factor Analysis (CFA). Int J Psychol Stud. 2016;8:p125.

2. Crenshaw AO, Christensen A, Baucom DH, Epstein NB, Baucom BRW. Revised scoring and improved reliability for the Communication Patterns Questionnaire. Psychol Assess. 2017;29:913–25.

3. Zimet GD, Dahlem NW, Zimet SG, Farley GK. The Multidimensional Scale of Perceived Social Support. J Pers Assess. US: Lawrence Erlbaum; 1988;52:30–41.

4. Eker D, Arkar H. Perceived social support: psychometric properties of the MSPSS in normal and pathological groups in a developing country. Soc Psychiatry Psychiatr Epidemiol. 1995;30:121–6.

5. Hampanda K, Abuogi L, Musoke P, Onono M, Helova A, Bukusi E, et al. Development of a Novel Scale to Measure Male Partner Involvement in the Prevention of Mother-to-Child Transmission of HIV in Kenya. AIDS Behav. 2020;24:291–303.

6. Hendrick SS. A Generic Measure of Relationship Satisfaction. J Marriage Fam. [Wiley, National Council on Family Relations]; 1988;50:93–8.

7. Hooley JM, Teasdale JD. Predictors of relapse in unipolar depressives: Expressed emotion, marital distress, and perceived criticism. J Abnorm Psychol. US: American Psychological Association; 1989;98:229–35.

8. Masland SR, Hooley JM. Perceived Criticism: A Research Update for Clinical Practitioners. Clin Psychol Sci Pract. 2015;22:211–22.

9. Pulerwitz J, Gortmaker SL, DeJong W. Measuring Sexual Relationship Power in HIV/STD Research. Sex Roles. 2000;42:637–60.

10. McMahon JM, Volpe EM, Klostermann K, Trabold N, Xue Y. A systematic review of the psychometric properties of the Sexual Relationship Power Scale in HIV/AIDS research. Arch Sex Behav. 2015;44:267–94.

11. Dunkle KL, Jewkes R, Nduna M, Jama N, Levin J, Sikweyiya Y, et al. Transactional sex and economic exchange with partners among young South African men in the rural Eastern Cape: prevalence, predictors, and associations with gender-based violence. Soc Sci Med 1982. 2007;65:1235–48.

12. Dunkle KL, Jewkes RK, Brown HC, Gray GE, McIntryre JA, Harlow SD. Gender-based violence, relationship power, and risk of HIV infection in women attending antenatal clinics in South Africa. The Lancet. 2004;363:1415–21.

13. STRAUS MA, HAMBY SL, BONEY-McCOY S, SUGARMAN DB. The Revised Conflict Tactics Scales (CTS2): Development and Preliminary Psychometric Data. J Fam Issues. SAGE Publications Inc; 1996;17:283–316.

14. Yoo H. Couple Intimacy and Relationship Satisfaction: A Comparison Study between Clinical and Community Couples [Internet]. The Ohio State University; 2013. Available from: http://rave.ohiolink.edu/etdc/view?acc_num=osu1374180064

15. Yoo H, Bartle-Haring S, Day RD, Gangamma R. Couple communication, emotional and sexual intimacy, and relationship satisfaction. J Sex Marital Ther. 2014;40:275–93.

16. Fraley RC, Heffernan ME, Vicary AM, Brumbaugh CC. The Experiences in Close Relationships-Relationship Structures questionnaire: a method for assessing attachment orientations across relationships. Psychol Assess. 2011;23:615–25.

17. Rusbult CE, Martz JM, Agnew CR. The Investment Model Scale: Measuring commitment level, satisfaction level, quality of alternatives, and investment size. Pers Relatsh. 1998;5:357–87.

18. Fifield J, O’Sullivan L, Kelvin EA, Mantell JE, Exner T, Ramjee G, et al. Social Support and Violence-prone Relationships as Predictors of Disclosure of HIV Status Among Newly Diagnosed HIV-positive South Africans. AIDS Behav. 2018;22:3287–95.

19. Aron A, Aron EN, Smollan D. Inclusion of Other in the Self Scale and the structure of interpersonal closeness. J Pers Soc Psychol. 1992;63:596–612.

20. Gächter S, Starmer C, Tufano F. Measuring the Closeness of Relationships: A Comprehensive Evaluation of the “Inclusion of the Other in the Self” Scale. PloS One. 2015;10:e0129478–e0129478.
